# The immune response to influenza in older humans: beyond immune senescence

**DOI:** 10.1186/s12979-020-00181-1

**Published:** 2020-05-07

**Authors:** Janet E. McElhaney, Chris P. Verschoor, Melissa K. Andrew, Laura Haynes, George A. Kuchel, Graham Pawelec

**Affiliations:** 1 Health Sciences North Research Institute, 41 Ramsey Lake Road, Sudbury, ON P3E 5J1 Canada; 2 Department of Medicine and Canadian Centre for Vaccinology, Dalhousie University, Halifax, NS Canada; 3 University of Connecticut Center on Aging, UConn Health Center, Farmington, CT USA; 4 Department of Immunology, University of Tübingen, Tübingen, Germany

**Keywords:** Influenza, Influenza vaccination, Hemagglutination inhibition antibody response, Broadly neutralizing antibodies, CD4 and CD8 T cell response, Cytokines, Granzyme B, Dendritic cells, Vaccine adjuvants

## Abstract

Despite widespread influenza vaccination programs, influenza remains a major cause of morbidity and mortality in older adults. Age-related changes in multiple aspects of the adaptive immune response to influenza have been well-documented including a decline in antibody responses to influenza vaccination and changes in the cell-mediated response associated with immune senescence. This review will focus on T cell responses to influenza and influenza vaccination in older adults, and how increasing frailty or coexistence of multiple (≥2) chronic conditions contributes to the loss of vaccine effectiveness for the prevention of hospitalization. Further, dysregulation of the production of pro- and anti-inflammatory mediators contributes to a decline in the generation of an effective CD8 T cell response needed to clear influenza virus from the lungs. Current influenza vaccines provide only a weak stimulus to this arm of the adaptive immune response and rely on re-stimulation of CD8 T cell memory related to prior exposure to influenza virus. Efforts to improve vaccine effectiveness in older adults will be fruitless until CD8 responses take center stage.

## Background

Despite widespread influenza vaccination programs and > 60% vaccination rates in the population age 65 years and older in many countries, influenza remains a serious threat to the health of older people. In the US, the over 65 population accounts for two-thirds of the 200,000 influenza-related hospitalizations independent of whether they are at high or low risk for serious complications of influenza [1], and older people make up 90% of the 36,000 deaths each year [2, 3]. Further, the length of hospital stay for older adults is almost 3-fold higher than 50–64 year olds and 6-fold higher than younger adults [1]. Recent global estimates of seasonal influenza-associated respiratory deaths have shown mortality rates of 50–100 per 100,000 in the over 75 population; non-respiratory causes of influenza-associated mortality require further investigation [4]. The aim of this article is to highlight those age-associated changes in the immune response to influenza vaccination that are due to multiple chronic conditions, their associated inflammatory effects, and increasing frailty. These changes go beyond what can be explained by immune senescence and are associated with the increased risk for serious complications of influenza including catastrophic disability. Our search of the existing literature in the preparation of this article included the terms: influenza, influenza vaccination, multiple chronic conditions or multi-morbidity, frailty, inflammation, disability, and humoral and cell-mediated immune responses. Current research efforts will require a deeper understanding of how the health of the ‘usual’ older person affects the cell-mediated immune response and how adjuvants may play a role in enhancing cellular immune mechanisms in the development of more effective influenza vaccines for older adults.

### Burden of influenza

Older adults are particularly susceptible to severe outcomes of influenza. This is especially true during seasons when A/H3N2 is the predominant circulating strain where dramatic increases in hospitalization rates occur in the population age 65 and older [5, 6]. Cardiovascular events are the most common extra-pulmonary complications of influenza (i.e., myocardial infarction, congestive heart failure, and strokes) with long-term consequences including cardiovascular disease and cognitive decline [7–9]. Dysregulated immune responses associated with multiple chronic conditions result from the chronically elevated levels of circulating inflammatory cytokines, often characterized as ‘inflammaging’ [10], and could very well be the mechanistic link to these complications of influenza. Specifically, the six leading causes of catastrophic disability including strokes [11, 12], congestive heart failure [13, 14], pneumonia and influenza [15–25], ischemic heart disease [8, 11, 26, 27], cancer and hip fracture [28, 29], have all been linked to influenza illness. Furthermore, 15% of older adults admitted with laboratory-confirmed influenza will experience catastrophic disability with a loss of independence in more than two basic self-care activities [30] and older adults are also vulnerable to diminished quality of life due to loss of independence following hospitalization with influenza [25]. In fact, influenza accounts for nearly 30% of all disability-adjusted life years lost to infectious disease [31], and influenza hospitalization is estimated to cost more than 2 billion dollars in Canada over the next decade [32]. Older adults, especially those living with chronic conditions and/or frailty, are highly susceptible to severe outcomes of influenza; however, the correlates of protection in this vulnerable population are not well investigated.

### Limitations of current influenza vaccines

Despite recent advances in the development of high-dose, adjuvanted, and subunit vaccines, there remain significant challenges to the development of more effective influenza vaccines for older adults, particularly for preventing the most serious complications. Influenza vaccines are annually formulated to stimulate strain-specific neutralizing antibody responses to the predicted circulating strains of influenza. These changes in the circulating strains are the result of antigenic drift wherein new strains emerge with human transmission due to the high mutation rates of the influenza A virus and the selection of strains with epitopes on the hemagglutinin (HA) head that are resistant to antibody binding. Vaccine strain mismatch may also result from the loss of glycosylation sites on the HA head in the process of adaptation of the vaccine strains for optimal growth in eggs. These neutralizing antibodies are stimulated in the response to influenza vaccination and thus prevent infection by binding to the HA head and neutralizing the virus to prevent virus entry into the host cell, so-called “sterilizing immunity”. Vaccine strain mismatch leads to a loss of strain-specific antibody binding to the critical protective epitopes surrounding the receptor-binding domain of the globular head of hemagglutinin. The hemagglutination inhibition (HAI) assay is the standard measure of strain-specific antibody titers and the rise in HAI antibody titers with vaccination is usually used to estimate vaccine efficacy in clinical trials. Much of the decline in vaccine efficacy with aging has been attributed to “immune senescence”, wherein the decline in the antibody response to influenza vaccination is attributed to age-related changes in B cell and T cell development and function (reviewed in [33–35]). These studies typically enroll healthy older adults who would be considered non-frail. Similarly, the older adult population represented in large Phase 3 clinical trials is generally healthier than those at an elevated risk and these trials are statistically powered to measure protection against mild to moderate influenza disease not requiring hospital admission. In this case, the antibody response to influenza vaccination may indeed be a good correlate of protection but may not predict “clinical protection” against severe influenza illness and hospitalization in the general population of older adults. A recent study of adults undergoing elective hip replacement has also shown the importance of the bone marrow environment in maintaining memory and effector cells and how the accumulation of both highly differentiated and terminally differentiated CD8 T cells is associated with declining serum antibody titers against diphtheria. This appears to be related to increased numbers of terminally differentiated CD8 T cells which negatively affect the maintenance of B cells and plasma cells, while increases in highly differentiated CD8 T cells lead to heightened pro-inflammatory cytokine levels and potentially toxic effects on B cell maintenance [36].

### Impact of frailty on outcomes of influenza

Frailty is a geriatric syndrome which is multifactorial in etiology, involves complex and dynamic interactions with the elements of biopsychosocial health, and reflects the loss of the adaptive capacity to respond to acute health challenges [37–39]. It has been generally recognized to be age-associated and common in older adults. The frailty phenotype (e.g. the Fried Frailty Score) provides a ternary (three category) view of frailty that considers weakness, slowness, inactivity, exhaustion and weight loss, and is associated with adverse outcomes including risk of mortality [40]. In contrast, the Frailty Index is a broader conceptualization [41], which relates to the accumulation of deficits across multiple domains of health and functional status [42], and is a more sensitive measure of the degree of frailty and strongly predicts mortality risk [43] and adverse outcomes [44]. For comparison, standard cutoffs for the frailty index are FI 0–0.1 = non-frail; > 0.1–0.21 = pre-frail; > 0.21–0.45 = frail; > 0.45 = most frail, and in community-based samples approximately 24% of older adults are frail [45].

Influenza illness is associated with increases in frailty, measured using a 40-item Frailty Index, among hospitalized older adults. Importantly, frailty is also an important confounder of vaccine effectiveness, and vaccine effectiveness declines with increasing frailty [46]. Our hospital-based influenza surveillance studies using a 40-item Frailty Index (FI) in adults age 65 years and older have found that older adult inpatients with laboratory-confirmed influenza have an average FI of 0.2 [30] indicating that this is a frail population [46] at high risk for catastrophic disability as a consequence of influenza illness. Further, recent studies suggest that the disabling outcomes of influenza [30] are not limited to the over 65 population. In the setting of critical illness, 50% of patients with pre-existing frailty are under age 65 [47]. Most of these patients are in the 50–64 year-old age range suggesting that multiple chronic conditions are a significant contributor to pre-existing frailty. More importantly, disability and mortality outcomes were dependent on the level of frailty as assessed by the Clinical Frailty Scale [48] and independent of chronological age. The ‘response’ to influenza vaccination can be measured as a serologic or cell-mediated immune responses but the impact of frailty on these responses has yet to be studied in a manner that captures its dynamic nature reflecting loss of resilience [49–51]. In addition, a protective response to vaccination may differ depending on the outcome studied; protection from mild to moderate illness may be very different from the response needed to prevent severe illness and the pulmonary and extrapulmonary complications of influenza.

In our community studies of the response to influenza vaccination in older adults, using the same 40-item Frailty Index, older adults on average have a Frailty Index of 0.1 (Non-frail are < 0.1) (unpublished observations). A systematic review of high-dose relative to standard-dose influenza vaccine trials, showed that the better antibody responses to high-dose influenza vaccine was independent of sex, age > 75 years old, frailty, and chronic conditions [52]. This is supported by individual studies from Singapore [53], Germany [49], and the U.S. [54], all of which reported that frailty did not have a significant effect on antibody titres following standard dose vaccination in adults 60 and over. Another study originating from the U.S. found the opposite, where antibody responses were significantly impaired in adults that were frail [55]. In apparent contrast, in our randomized trial of high-dose vs. standard-dose vaccine, increasing frailty increased absolute titres post-vaccination as well as the likelihood of being a vaccine responder (i.e., a four-fold rise in HAI antibody titers) [56]. This was similarly observed by Moehling and colleagues, specifically in adults between 50 and 65 years old [51]. A systematic review of MF-59 adjuvanted vs. unadjuvanted influenza vaccine also showed an enhanced antibody response with the adjuvanted formulation in older adults but an analysis of the effects of sex, older age, frailty or chronic conditions was not performed [57]. The disagreement within the literature is perplexing, and at the moment, the underlying reasons are not clear. One possibility is that the relationship between vaccine antibody responses and frailty (if one does indeed exist) depends on the manner in which frailty is measured and also the manner in which changes to antibody titres are classified as indicating responder status. Thus, the studies referred to above, including our own, generally use either the Frailty Phenotype [40] or Frailty Index [42] approach, and treat the measure as either a categorical or continuous manner. Another possibility is that the frailty relationship depends on the vaccine formulation, both the dose and strains targeted. Findings from our clinical trial, which included more than 600 participants, suggest that all of these factors play an important role and should be considered more closely in future studies. Further, these factors may be associated with a loss of reliability of the antibody response to influenza vaccination as a measure of vaccine efficacy and correlate of protection in more frail individuals.

Test-negative case control design studies are another way to investigate the effect of frailty on laboratory-confirmed influenza-related hospitalizations, an outcome of vaccination that cannot be captured in sufficient numbers in clinical trials to demonstrate the enhanced benefit of newer vaccines. All patients admitted with an acute respiratory illness are included in these studies where those patients with laboratory-confirmed influenza are compared to those patients who are test-negative for influenza [58]. These studies provide annual estimates of influenza vaccine effectiveness for the prevention of hospitalization in Canada through the Serious Outcomes Surveillance (SOS) Network of the Canadian Immunization Research Network [46, 59–61]. More recently, a test-negative case control design incorporating the 40-item Frailty Index in SOS Network studies has demonstrated the importance of considering the level of frailty as a major contributor to the decline in influenza vaccine effectiveness in older adults [46]. This is supported by work of Petrie and colleagues [62], which showed that vaccine efficiency in frail older adults is 17%, as compared to 48% for all adults over 65 years old. These results also suggest that frailty is a major contributor to the risk for serious complications of influenza, even in vaccinated older adults. Understanding the determinants of T-cell mediated clinical protection against serious influenza illness, as a paradigm shift in in the scientific community and US Food and Drug Administration approval pathways remains a major challenge [63].

### Cell-mediated immune protection against influenza

When antibodies fail to protect against influenza infection by providing sterilizing immunity, “clinical protection” relies on cell-mediated immune mechanisms to the clear the virus from infected host cells and prevent the pulmonary and extrapulmonary complications of influenza. Previous studies have shown that heterosubtypic immunity (i.e., cross-protective across multiple influenza A strains) induced in the mucosal tissue of the respiratory tract by influenza infection is mediated by CD8 cytotoxic T lymphocyte (CTL) responses [64] and by broadly non-neutralizing antibodies (bNA) through antibody-dependent phagocytosis by alveolar macrophages [65]. Influenza infection stimulates both naïve and memory B cells and CD4 and CD8 T cells (reviewed in [66]), and is required to establish influenza-specific CD8 T cell immunologic memory. In contrast, current standard seasonal influenza vaccination (inactivated virus) primarily stimulates B cells and CD4 T cells, while the CD8 T cell response relies on re-stimulation of immunologic memory from prior exposure to the virus – inactivated influenza vaccines provide only a weak stimulus to virus-specific CD8 T cells. In young adults with low HAI titers, the CTL response to influenza infection has been shown to correlate with protection against influenza illness [67]. More recently, increased levels of interferon (IFN)γ-producing CD4 T helper type 1 (Th1)) and CD8 CTL cells responding to the conserved internal proteins of influenza virus, matrix (M1) and nucleoprotein (NP), have been shown to correlate with protection against influenza A strains [68, 69]. Importantly, these internal proteins are shared across the A/H3N2 and A/H1N1 subtypes of influenza A, thus providing heterosubtypic immunity whereby the response to these internal proteins provides protection across multiple strains of influenza. We have shown in multiple studies that the T cell response following influenza vaccination with split-virus vaccine (SVV) formulations using ex vivo influenza challenge of peripheral blood mononuclear cells (PBMC) correlates with protection in older adults [70–72]. In contrast, a randomized clinical trial of four subunit vaccines (containing no internal proteins) demonstrated an enhanced HAI antibody response to the M59-adjuvanted formulation over the unadjuvanted vaccines [73], but there was no difference in the T-cell response across the four vaccines [74]. Although antibody-dependent phagocytosis as a correlate of protection against influenza illness in humans has not been studied, observations from studies of macrophage function in the context of aging suggest these investigations may be warranted.

### Role of innate immune cells in influenza infection

Comparisons of young and old mice have yielded conflicting results, possibly arising from discrepancies in experimental design, macrophage type studied, and exactly how “old” is defined [75], but at least two studies have shown that peritoneal phagocytic function is impaired with age [76]. Similarly, many studies have revealed age-associated differences in macrophage functionality in humans (reviewed in [77]). We are unaware of any studies that have investigated changes in human macrophage phagocytic function with age, but a relatively recent study did find that the uptake of opsonized bacteria was significantly reduced in CD14+ monocytes from older adults [78]. This becomes important as influenza infection has been shown to induce inflammation causing lung injury and life-threatening pneumonia, either primary viral pneumonia or secondary bacterial infection [79]. This acute inflammation in response to influenza infection is mediated by lung monocytes and monocyte-derived dendritic cells that are involved in the detection and clearance of influenza virus and influenza pathogenesis in the lungs [80]. These innate immune cells are not only susceptible to influenza-induced apoptotic and necrotic cell death [81], influenza infection has a direct effect on monocytes which results in the downregulation of Th17-mediated immunity necessary for protection against secondary pneumococcal pneumonia [82]. This effect of influenza infection on the monocyte function necessary for the clearance of pneumococcus has recently been demonstrated in a human challenge model [83].

Natural killer (NK) cells have also been shown in mouse models to contribute to the control of influenza during the early stage of infection through granule-mediated (granzyme B and perforin) killing of virus-infected cells [84–87], but can also be killed themselves by the influenza virus. Aged mice show a decline in the numbers and function of NK cells with more immature and less fully mature NK cells. These age-related changes may impair the ability of NK cells to contribute to a strong antiviral response during the early phase of influenza infection [88]. In humans, fatal influenza cases show low numbers of NK cells in the lungs [89, 90] and this has been associated with the effects of influenza infection in NK cells which inhibits the cytolytic function and cytokine and chemokine secretion [91]. However, there is limited knowledge as to how these age-related changes found in mouse models or in fatal cases of human influenza in young adults relate to outcomes of influenza in older adults [92].

### Toll-like receptor agonists as influenza vaccine adjuvants

Appropriate selection of TLR agonists which stimulate dendritic cells (DC) is needed to improve the CD8 T cell response to influenza challenge in older adults. The primary targets for TLR agonists are the TLR on myeloid (mDC) and plasmacytoid (pDC) dendritic cells but studies have shown that the age-related decline in the engagement of TLR1/2, TLR2/6, TLR3, TLR5, and TLR8 in mDCs and TLR7 and TLR9 in pDCs, may be mediated by dysregulated cytokine production [93]. These results are consistent with our unpublished experiments in which we screened different TLR agonists and found that glucopyranosyl lipid adjuvant formulated in a stable emulsion (GLA-SE, a TLR4 agonist) provided a superior stimulus in terms of cytokine production and that the cytokine response (IL-6, TNF, IL-12) was mainly in mDC [94]. More recent global analyses using commercially-available TLR agonists such as lipopolysaccharide (LPS) to stimulate TLR4 have also demonstrated age-related changes in innate immune responses after stimulation with different TLR agonists [95]. Using frozen PBMC in these experiments, it was concluded that the delay in the response to TLR agonists in older adults is not due to differences in numbers of DC and monocytes but rather is due to an impairment of downstream signalling events in monocytes and DC. Our studies have shown that functional mDC do not survive freezing very well and that immune cells may be stripped of important receptors after cryopreservation, both of which could have contributed to the above findings using frozen PBMC. In a comparative systems analysis of the molecular signatures of clinically-tested, non-TLR vaccine adjuvants, GLA-SE was shown to provide better stimulation of different B and T cell subsets [96]. Both components of the GLA/SE adjuvant were found to be required to achieve optimal responses in both arms of the adaptive immune response: specifically, SE for neutralizing antibodies and GLA for induction of T cell responses [97].

Vaccine adjuvants including TLR agonists have been used to enhance Th and CTL responses to influenza vaccination. Different vaccine adjuvants can stimulate Th1 (e.g. MF59 [squalene based] + CpG [TLR9 agonist]) vs. Th2 (e.g. MF59 alone) responses to influenza vaccination according to the relative degree of Th1 production of IFNγ vs. Th2 production of IL-5; these actions contribute to antibody and cell-mediated protection against influenza [98]. However, mice that received MF59 + CpG adjuvanted influenza vaccine were better protected against influenza illness with less weight loss and better survival compared to mice that received MF59-adjuvanted vaccine. In addition, these mice showed better resistance to a subsequent bacterial superinfection. It remains to be determined whether vaccine-induced T-helper cells contributed to viral clearance via secretion of immune-activating cytokines and/or via direct effector functions. In aged mice, we have shown that the addition of inflammatory cytokines (IL-1, IL-6 and TNF or IL-2 plus IL-6 can reverse age-related defects in Th1 cytokine production, proliferation and function [99]. In contrast, human PBMC cultured with influenza vaccine and inflammatory cytokines (IL-1, IL-6 and TNF) showed a suppressed T cell response to a subsequent influenza challenge [94]. However, the addition of IL-2 and IL-6 to influenza H3N2-challenged PBMC cultures restored the proliferative response and function of influenza-specific CD8 CTL in aged mice and humans to that of their young counterparts [100]. Importantly, our studies have shown that the CTL response to ex vivo influenza challenge is dependent on the presence of CD4 T cells in the PBMC cultures [100, 101].

### T cell correlates of protection

To the best of our knowledge, we have published the only prospective studies of T cell correlates of protection against influenza in vaccinated older adults. In multiple studies, we have shown in PBMC that the ratio of IFNγ to interleukin (IL)-10 (IFNγ:IL10) in supernatants of stimulated PBMC and biological activity of the cytolytic mediator, granzyme B (GrB), in lysates of ex vivo influenza challenged PBMC correlate with protection against influenza illness [70, 71] and disease severity in older adults [72]. This GrB activity at 4-weeks post-vaccination as a correlate of protection can be demonstrated even among frail older adults [102]. Importantly, we have shown that stimulation of immunologic memory during an influenza infection can subsequently be restimulated by influenza vaccination and these flu+ cases show an enhanced response to vaccination compared to non-infected older adults [71]. These results suggest that the decline in the CTL response to influenza vaccination with aging is a limitation of current vaccines NOT a compromised immune system per se.

### The role of influenza internal proteins and adjuvants in new vaccine development

Novel strategies for stimulating CD8 CTL responses have long been a priority for new vaccine development to provide heterosubtypic immunity across serologically distinct strains of virus [64, 94, 103–105]. Since the current inactivated influenza vaccine formulations provide only a weak stimulus to CD8 T cell responses, adjuvanted formulations that enhance T cell help and antigen presentation to CD8 T cells are being pursued. Because the response to influenza M1 and NP, correlates with protection in both CD4 [68] and CD8 T cells [69], these internal proteins will be a necessary component of new vaccines for older adults. These internal proteins are absent in influenza subunit vaccines (HA +/− neuraminidase), so alternative mechanisms including antibody-mediated protection [106] may explain the enhanced protection of adjuvanted [57] and recombinant HA [107] formulations in older adults. A large randomized clinical trial of an AS03-adjuvanted influenza vaccine in older adults also showed enhanced protection against the influenza A/H3N2 vaccine strain compared to the unadjuvanted formulation [108], and this enhanced protection was comparable to that found in the randomized trial of high-dose influenza vaccine [109].

The importance of including influenza internal viral proteins such as M1 and NP in vaccines has been highlighted in experiments in the mouse model showing that sequential immunization with whole-inactivated influenza vaccine (containing influenza internal proteins), but NOT a subunit vaccine (no internal proteins), could alleviate the severity of infection with antigenically drifted viruses from the vaccine strains and potentially improve protection to unpredictable seasonal infection [110]. Importantly, depletion of T cells prior to challenge revealed that CD8 T cells, but not CD4 T cells, contributed to cross-protection. However, CD4 T cells are needed to provide cytokine-mediated ‘help’ to optimize the generation of a CD8 T cell response. Similarly, human PBMC stimulated with different inactivated influenza vaccines showed statistically significant differences in the numbers of activated IFNγ-producing cells depending on the amount of internal proteins contained in the vaccine [111]. These results are consistent with a clinical study showing that vaccine effectiveness for the prevention of hospitalization in older adults was 77.8% in recipients of SVV (containing M1 and NP) but only 44.2% in subunit vaccine (surface glycoproteins only) recipients, giving a difference in vaccine effectiveness of 33.5% [112]. A randomized study of four subunit vaccines (which did not contain M1 and NP) confirmed that while an MF59 adjuvant could stimulate higher antibody responses compared to unadjuvanted influenza vaccines [73], we showed that there was no difference in T cell responses to adjuvanted vs. non-adjuvanted subunit formulations [74]. Taken together, these results suggest that M1 and NP will need to be included in vaccine formulations designed to prevent the serious complications of influenza in older adults, particularly those who are frail.

Importantly, we have shown in in vitro experiments that there was an increase in the IFNγ:IL-10 ratio and GrB activity in response to influenza challenge when the TLR4 agonist GLA-SE was combined with SVV. This response appears to be mediated by a 10-fold reduction in IL-10 levels when older adult PBMC are stimulated with GLA-SE plus SVV compared to SVV alone prior to influenza challenge [94]. Our unpublished results show that SVV stimulates IL-2 production and GLA-SE stimulates IL-6 production in these PBMC cultures. Furthermore, we have shown that the combined effects of IL-2 and IL-6 as a supplement in ex vivo influenza-challenged PBMC restores the CD8 T cell response to that seen in younger adults; the problem is that older adults have a lower frequency of CD8 T cells at baseline [100]. These results suggest a mechanism that can be targeted using vaccine adjuvants to develop more effective influenza vaccines for older adults.

## Conclusions

Current research efforts to support the development of new influenza vaccines have largely focused on hemagglutination inhibition antibody responses. Defining those changes that occur with immune senescence requires studies of relatively healthy older adults who are not generally representative of the over 65 population with multiple chronic conditions, particularly those age 75 years and older. While antibody responses to influenza vaccination may predict protection against influenza infection in community-dwelling older adults, it does not explain the significant increases in their hospitalization rates that occur during influenza seasons where A/H3N2 (relative to A/H1N1 and B strains) is the predominant circulating strain. Immunologic priming with the first exposure to influenza in childhood (i.e. A/H1N1 for the current older adult cohort) may explain the low hospitalization and death rates during A/H1N1 years, with only the most frail being susceptible to the serious complications of influenza. Further, dysregulated immune responses characterized as ‘inflammaging’ in the context of multiple chronic conditions and frailty appear to be the mechanistic link to these complications, and go beyond those changes in lymphocyte development and function attributed to immunosenescence. More studies are needed in older adults with multiple chronic conditions to determine how varying levels of frailty lead to the loss of adaptive capacity or resilience and are reflected in multiple measures of the interaction of innate and adaptive immune function. Current influenza vaccines are poor stimulators of the heterosubtypic cell-mediated immunity needed to prevent the serious complications of influenza. We propose that there is a requirement to translate new insights into how cell-mediated immune responses to influenza A/H3N2 strains can be enhanced in the design of new influenza vaccines. To address this unmet need, we must first understand the interaction between circulating proteins associated with inflammatory processes, the phenotype and function of different immune cells subsets responding to influenza challenge, and prove that cell-mediated immune correlates of protection are valid in older adults. It will also be necessary to better understand how broadly-neutralizing antibodies contribute to heterosubtypic immunity. Finally, our in vitro system using older adult PBMC to simulate the effect of different vaccine/adjuvants on the response to influenza challenge using our established correlates of protection, and our choice of adjuvants currently in clinical development, serves as a model for pre-clinical testing and an accelerated pathway through the clinical development pipeline.

## Data Availability

Not applicable.

## Abbreviations

bNA: Broadly non-neutralizing antibodies
CTL: Cytotoxic T lymphocytes
DC: Dendritic cells; myeloid (mDC) and plasmacytoid (pDC)
GrB: Granzyme B
HAI: Hemagglutination inhibition
IFN: Interferon
IL: Interleukin
M1: Influenza matrix protein
NP: Influenza nucleoprotein
PBMC: Peripheral blood mononuclear cells
SOS: Serious Outcomes Surveillance Network
SVV: Split-virus influenza vaccines
Th1: T helper type 1
Th2: T helper type 2
TLR: Toll-like receptor
TNF: Tumour-necrosis factor
